# Commentary: Correlation between different levels of thyroid autoantibodies and immune checkpoint inhibitor-associated thyroid dysfunction

**DOI:** 10.3389/fendo.2025.1686015

**Published:** 2025-10-07

**Authors:** Dongdong Zhang, Ran Jing, Ziwei Chen, Pei Zhang, Ming Cai

**Affiliations:** Department of Thyroid Oncology, Chongqing Key Laboratory of Translational Research for Cancer Metastasis and Individualized Treatment, Chongqing University Cancer Hospital, Chongqing, China

**Keywords:** neoplasms, thyroid gland, immune checkpoint inhibitors, immune-related adverse events, anti-thyroid peroxidase antibody

## Introduction

The study by Jiao et al. (2025) investigates the critical association between pre-existing thyroid autoantibodies (TPOAb, TRAb, TgAb) and immune-related thyroid adverse events (irAEs) in cancer patients receiving immune checkpoint inhibitors (ICIs) (1). By retrospectively analyzing 316 patients, the authors identify TPOAb and TRAb positivity as significant risk factors (OR >6.9) for thyroid dysfunction. This work underscores the potential of baseline antibody screening for risk stratification, aligning with emerging evidence on ICI-induced autoimmunity. However, methodological limitations and unexplored confounders necessitate cautious interpretation of its clinical implications.

## Subsections relevant for the subject

First, the study’s retrospective analysis at Qingdao Chengyang District People’s Hospital (2018—2023) risks selection bias and geographic homogeneity. All patients were Chinese, and 68% had lung cancer—a distribution inconsistent with global ICI trial cohorts (2). This may overestimate autoantibody prevalence (28.5% in irAEs group vs. 5—15% in multinational reports). Additionally, the exclusion of prior thyroid dysfunction could artificially amplify associations between antibodies and irAEs. External validation in diverse populations is essential.

Second, the authors report divergent antibody profiles: TRAb dominated thyrotoxicosis, while TPOAb correlated with hypothyroidism. Yet, they omit cellular immunity data (e.g., T-cell subsets, cytokines), which are pivotal in ICI-induced thyroiditis (3). Murine models confirm CD4+ T-cell infiltration drives thyroid damage during anti-PD-1 therapy, and human studies implicate thyroglobulin-specific CD8+ T cells in Hashimoto’s-like injury (4). Without integrating immunophenotyping, the study cannot elucidate whether antibodies are causal or bystanders in irAEs pathogenesis.

Third, Critical technical details are missing: 1. Timing variability: Antibody tests occurred “≥4 weeks post-ICI,” but irAEs onset ranges from days to months. Fluctuations in titers during treatment were unmonitored. 2. Assay heterogeneity: The study omits detection methods, despite known inter-assay variability in antibody quantification. 3. Threshold justification: TRAb positivity (threshold: >1.75 IU/L) was rare (1.26% in irAEs group), raising questions about statistical power for regression analysis (OR = 6.98).

Fourth, patients received mixed ICI regimens (Tislelizumab, Pembrolizumab, Xindilimab). Combination CTLA-4/PD-1 therapy (7.59% of irAEs group) has a 3-fold higher thyroid irAE risk than PD-1 monotherapy, yet subgroup analysis was neglected. Prior studies show PD-1 inhibitors preferentially induce hypothyroidism, while CTLA-4 blockers cause thyrotoxicosis (5, 6). Merging these distinct mechanisms may confound antibody correlations—especially for TRAb (Graves-like toxicity) vs. TPOAb (Hashimoto-like injury).

Finally, gynecologic cancers constituted 12% of irAEs cases but only 1.9% of controls. Endometrial and ovarian cancers exhibit high tumor mutational burden (TMB) and PD-L1 expression, which independently increase irAE susceptibility. The study adjusted for tumor type in regression but did not explore how tumor-specific immunity interacts with thyroid antibodies. This is a missed opportunity to refine risk stratification.

## Discussion

This study provides valuable clinical evidence supporting baseline thyroid antibody screening (particularly TPOAb/TRAb) for ICI recipients. However, the retrospective nature of this study and its insufficient mechanistic exploration constrain translational applicability. Future research should integrate multi-omics profiling to elucidate causal relationships between autoantibodies and thyroid irAEs, implement standardized longitudinal antibody monitoring during ICI therapy to capture dynamic immune changes, and develop personalized risk algorithms through stratification by ICI class and tumor immunophenotype. Critically, prospective interventional trials evaluating preemptive strategies—such as prophylactic thyroid hormone replacement for TPOAb-positive patients—are warranted to mitigate clinical morbidity. The clinical impact of these findings and remarks extends beyond specialist circles. As ICIs are increasingly used across a spectrum of malignancies, the management of their toxicities has become a multidisciplinary endeavor. The recognition that pre-existing autoimmunity—detectable by simple serological tests—is a key risk factor has direct implications for a broad audience of clinicians, including internists, general practitioners, and other specialists who may be the first to encounter non-specific symptoms of thyroid dysfunction in these patients. Implementing the proposed strategy of baseline risk stratification empowers the entire care team to enhance vigilance, facilitate earlier diagnosis and endocrinology referral, and improve patient counseling. This proactive approach is essential not only for mitigating morbidity but also for safeguarding the continuity of potentially life-saving anticancer therapy, thereby maximizing overall patient safety and treatment success.
